# Colorectal Cancer and Probiotics: Are Bugs Really Drugs? †

**DOI:** 10.3390/cancers12051162

**Published:** 2020-05-05

**Authors:** Purushottam Lamichhane, Morgan Maiolini, Omar Alnafoosi, Sedra Hussein, Hasan Alnafoosi, Stewart Umbela, Tayanna Richardson, Nevien Alla, Narottam Lamichhane, Bobban Subhadra, Rahul R. Deshmukh

**Affiliations:** 1 LECOM School of Dental Medicine, 4800 Lakewood Ranch Blvd, Bradenton, FL 34211, USA; plamichhane@lecom.edu; 2 LECOM School of Pharmacy, 5000 Lakewood Ranch Blvd, Bradenton, FL 34211, USA; MMaiolini21274@rx.lecom.edu (M.M.); OAlnafoosi79536@rx.lecom.edu (O.A.); HAlnafoosi89566@rx.lecom.edu (H.A.); Stewart.Umbela@rx.lecom.edu (S.U.); trichardson@lecom.edu (T.R.); NAlla92956@rx.lecom.edu (N.A.); 3 Upstate Medical University, 750 East Adams Street, Syracuse, NY 13210, USA; Husseins@upstate.edu; 4 Department of Radiation Oncology, University of Maryland School of Medicine, Baltimore, MD 21201, USA; NaruLamichhane@umm.edu; 5 BIOM Pharmaceuticals, 2203 Industrial Blvd, Sarasota, FL 34234, USA; bbobban@gmail.com

**Keywords:** colorectal cancer, chemoprevention, probiotics, gut microflora, bacteria

## Abstract

Colorectal cancer (CRC) is one of the most common types of cancer worldwide. There are many factors that predispose a patient to the disease such as age, family history, ethnicity, and lifestyle. There are different genetic factors and diseases that also increase a person’s risk for developing CRC. Studies have found associations between gut microbiome and the risk for developing versus protection against CRC. Normal gut microbiome aid in daily functions of the human body such as absorption, metabolism, detoxification, and regulation of inflammation. While some species of bacteria prevent CRC development and aid in therapeutic responses to various treatment regiments, other species seem to promote CRC pathogenesis. In this regard, many studies have been conducted to not only understand the biology behind these opposing different bacterial species; but also to determine if supplementation of these tumor opposing bacterial species as probiotics lends toward decreased risk of CRC development and improved therapeutic responses in patients with CRC. In this literature review, we aim to discuss the basics on colorectal cancer (epidemiology, risk factors, targets, treatments), discuss associations between different bacterial strains and CRC, and discuss probiotics and their roles in CRC prevention and treatment.

## 1. Introduction

### 1.1. Epidemiology

Colorectal cancer (CRC) is the third most commonly diagnosed cancer, excluding skin cancer, and the third most deadly cancer in the United States [1]. The estimated new cases for 2020 are 147,950 and it is estimated to cause close to 53,200 deaths in both genders in 2020 [2]. Fortunately, the incidence of colorectal cancer has steadily declined from 53.3 per 100,000 individuals in 2000 to 35.7 per 100,000 individuals in 2017. At the same time period, the death rate from this cancer has modestly declined from 20.7 to 13.5 deaths per 100,000 individuals [2]. The 5-year survival, however, has remained unchanged at 65.0% in 2000 and 65.7% in 2012, with the cumulative rate of 64.6% from 2010–2016 [2]. Additionally, while incidence of colorectal cancer has declined in individuals older than 50 years from 2009 to 2013, it has increased in individuals younger than 50 years old. The death rates, however, have decreased in adults younger than 50 years old, while they have increased in individuals older than 50 years of age. Currently, it is not completely understood why these trends are occurring [3,4]. 

Incidence of CRC also varies between different ethnic groups and socioeconomic status. Lower socioeconomic status has been found to be associated with a higher prevalence of risk factors for CRC such as obesity, smoking, and unhealthy diet. Additionally, patients with lower socioeconomic status may have inadequate access or affordable access to screening methods and early treatments [4]. There are also geographical disparities in the incidence rates of CRC. In the USA, it is more prevalent in Southern and Midwestern states. This is both due to the difference in demographics, but also other localized factors such as laws/regulations, travel distances to access medical services, and socioeconomic issues [4]. The breakdown of statistics, trends in incidences and mortality, and relative risks for CRC are well documented in detail by the American Cancer Society in its “Colorectal Cancer Facts & Figures 2017–2019” document [4]. Globally, the distribution also has variations. In Northern and Western Europe, rates of colon cancer have been stabilizing. However, Japan, Singapore, and Eastern Europe have seen rapidly increasing rates of colon cancer since the 1970s. Rates of colon cancer have always traditionally been lowest in Africa and other low-income regions of the world [5].

Incidence of right-sided (proximal colon) colon tumors has increased over the past decade while the incidence of left-sided (distal) colon tumors has decreased [6]. Right-sided colon cancer is when the neoplasm is found in the cecum and ascending colon up to the hepatic flexure. Left-sided colon cancer is in the splenic flexure or surrounding including the rectum [6]. This can be explained by the fact that screening is better at detecting left-sided tumors compared to right-sided ones. This is due to the development, location, and morphology of the tumor growths. Routine screenings, and removal of adenomatous polyps, when found, have decreased the incidence of left-sided colon cancer significantly. In addition to difficulty in detecting, right-sided tumors are also more difficult to treat due to region of growth and a greater number of genetic mutations; these tumors, however, seem to respond better to immunotherapies (including but not limited to ipilimumab, tremelimumab, nivolumab) [7].

### 1.2. Colon Cancer

#### 1.2.1. Risk Factors and Potential Causes of Colon Cancer Carcinogenesis 

There does not appear to be any specific, single known cause of colorectal cancer. There are numerous risk factors that are linked to the development of colorectal cancer. The mechanisms by which these risk factors cause colorectal cancer carcinogenesis are poorly understood. Lynch Syndrome and familial adenomatous polyposis are perhaps the only exceptions to this rule as they are genetic conditions, but they do not account for the majority of colorectal cancer incidence globally [3,4]. Keeping this in mind, the following list is an overview of some of the most prominent risk factors and the associated mechanisms for the development of colorectal cancer:

##### Bacteria

Certain bacterial strains and dysbiosis of gut microbiota have been associated with the development of colorectal cancer. Species such as *Enterococcus faecalis, Shigella, Bacteroides fragilis,* and more can produce toxins and superoxide (SO) agents; and increase host inflammatory responses; hence driving the process of colorectal carcinogenesis [8]. Increased abundance of enterotoxigenic *Bacteroides fragilis* (ETBF) has been found in early-stage lesions of colorectal neoplasia [9] and stool samples from colorectal cancer patients [10]. Additionally, exposure of colon mucosa to *Bacteroides fragilis* toxins has been suggested to be a risk factor for CRC development [11]. Elsewhere, Chung et al. showed that *B. fragilis* toxin drives tumorigenesis of colonic epithelial cells through mechanisms that are dependent on interleukin-17 (IL-17) and signal-transducer-and-activator-of-transcription 3 (STAT3) activity [12]. In one preclinical study, however, Lee et al. showed that *B. fragilis* colonization protected mice from colitis-associated CRC through a mechanism dependent on polysaccharide A production and toll-like receptor 2 (TLR2) signaling and associated with inhibition of C–C motif chemokine receptor 5 (CCR5) in the colon [13]. *Streptococcus bovis,* specifically *Streptococcus gallolyticus*, is another bacterium that correlates with increased rates of CRC [14]. It has been shown that *Streptococcus gallolyticus* may promote inflammation-driven development or progression of CRC through mechanisms dependent, in part, on interleukin-1 (IL-1), interleukin-8 (IL-8), and cyclooxygenase 2 (COX-2) signals [14,15]. In the analysis of colorectal cancer tissues, it was identified that *S. bovis/gallolyticus* promotes colorectal carcinogenesis through inflammatory mechanisms dependent, in part, on IL-1, IL-8, and COX-2 signals [14]. In a model of azoxymethane-treated rats, cell wall components from *Streptococcus bovis* were shown to promote pre-neoplastic lesions through mechanisms dependent on IL-8 and COX-2, and the activation of MAPK signals [15]. Many other bacterial strains such as *Enterococcus faecalis*, *Shigella*, and *Escherichia coli* (NC101) have also been shown to drive CRC development [8]. *Fusobacterium nucleatum* is another bacterium whose concentrations in tissue and feces is found at higher levels at later stages of CRC [16]. *F. nucleatum* mediated promotion of CRC may involve FadA adhesion mediated modulation of E-cadherin/β-catenin signaling [17] and Fap2 binding to inhibitory receptor TIGIT (T cell immunoreceptor with Ig and ITIM domains) and subsequent inhibition of NK cell mediated cytotoxicity and T-cell activity [18]. Additional mechanisms of *F. nucleatum* mediated colorectal carcinogenesis have been reviewed by Shang and Liu [19]. Some of these mechanisms include miR-21 expression, hyperactivation of NF-κB, suppression of T-cell activity, and attraction of myeloid derived suppressor cells (MDSCs) [19]. It is generally agreed that bacterial causes to CRC are normally due to a variety of species rather than a solo bacterium [19,20,21]. Some of the bacteria and mechanisms associated with CRC or promotion of CRC are summarized in Table 1.

##### Bacteria and Protection against Colorectal Cancer

While it is evident that some bacteria associate with or drive CRC development and progression, others may have a protective role; hence highlighting the role of dysbiosis in CRC. Lactic acid producing bacteria (LAB) have been shown to confer protection against CRC [22]. *Bifidobacterium*, a LAB and part of normal human microflora, has been reported to have anti-tumor properties [23]. In a human study, it was found that intake of yogurt containing *Bifidobacterium longum* resulted in increased amounts of short-chain fatty acids (SCFAs) and decreased *Bacteroides fragilis* enterotoxin in feces. The same study showed that short-chain fatty acids such as butyric acid, isobutyric acid, and acetic acid had growth inhibitory activity against colon cancer cell lines in vitro [24]. Animal studies have also shown benefits of different bacteria. Mechanisms by which bacteria confer protection against CRC include competition against pathogenic bacteria, metabolic functions, and genetic modulations [22]. In a colitis-induced murine model of CRC, administration of *Bifidobacterium longum* resulted in increased expression of tumor suppressor micro-RNAs (miRs) miR-145, and miR-15a, decreased expression of miR-146a (which regulates expression of interleukins 1β and 6), decreased nuclear factor kappa-light-chain-enhancer of activated B cell (NF-κB) activation, and resulted in decreased aberrant crypt foci numbers [25]. In another murine study, *Bifidobacterium longum* has also been reported to suppress food mutagen (2-Amino-3-methylimidazo(4,5-f)quinoline) induced colon cancer incidence [26]. Many other LABs have been shown to have protective roles against CRC. These include *Lactobacillus acidophilus, Streptococcus thermophilus, Lactobacillus casei, Lactobacillus salivarius, Lactobacillus plantarum,* and *Lactobacillus rhamnosus* [22,27]. Mechanisms of inhibition of CRC initiation and progression employed by LAB include induction of apoptosis of tumor cells, metabolic antioxidant activity, activation of anti-tumor immune effectors, and anti-tumorigenic epigenetic modifications through metabolic products such as SCFAs [22,27]. Some of the bacteria and mechanisms associated with CRC or promotion of CRC are summarized in Table 2.

In the context of immunotherapy, some bacterial species such as *Bifidobacterium* have been shown to promote anti-tumor immunity and also correlate with the efficacy of programmed death ligand-1 (PD-L1) checkpoint blockade [28]. Interestingly, *B. fragilis*, which is shown to promote CRC carcinogenesis, was found to promote efficacy of cytotoxic T-lymphocyte-associated protein 4 (CTLA-4) blockade in melanoma patients [29]. Specifically, presence of *B. fragilis* specific T cell responses were associated with improved efficacy of CTLA-4 blockade [29]. These reports suggest that presence of some bacterial species, their components, and existing or induced immune responses against them might determine the efficacy of some checkpoint inhibitor therapies. These results also highlight, however, that associations alone without information on mechanisms may not be enough to make conclusions about the definitive role of these bacteria in promotion or inhibition of CRC initiation and progression.

##### Genetic Factors

Many cases of colorectal cancers occur in people with no family history of the disease. However, individuals with one first-degree relative under 60, or greater than two first-degree relatives of any age, that have had colorectal cancer or adenomatous polyps, are considered to have a significantly higher risk [30,31]. Approximately 20% of CRC patients have family members that are also affected by this disease. It is estimated that 5–10% of colorectal cancers are inherited [32]. A single gene, a combination of genes, or a combination of genetic and environmental factors can contribute to familial colorectal cancer. Typically, these families have one or two members with a history of colorectal cancer or pre-cancerous polyps. Mutations in several genes associated with hereditary colorectal cancer have been identified [31,32]. Some of these include mutations in the genes MLH1, MSH2, MSH6, APC, EPCAM, MUTYH, and PMS2. All of these genes are inherited in an autosomal dominant manner except for MUTYH, whose mode of inheritance is recessive [31,32]. The genetic causes of hereditary colorectal cancer syndromes, familial adenomatous polyposis (FAP), Lynch Syndrome, and hereditary nonpolyposis colorectal cancer (HNPCC) have been identified [31,32,33].

Lynch Syndrome is an autosomal dominant condition that comprises approximately 3–5% of colorectal cancers. Those with Lynch Syndrome have mutations in mismatch repair genes and hence have an increased propensity to form adenomas and a 20–80% likelihood of developing certain types of cancer during their lifetime [34]. With this disease, cancer tends to occur at younger ages, and at more proximal locations, rather than sporadically forming. This is in part due to the early formation of polyps and earlier gene mutations in cells [35]. The underlying genetic causes are mutations in DNA causing dysfunction of mismatch repair enzymes. Currently, mutations in four major genes (*MLH1, MSH2, MSH6, PMS2*), involved in mismatch repair functionality, have been associated with CRC. Each of these genes incurs a different probability of developing colon cancer [35,36,37]. 

Familial adenomatous polyposis (FAP) is a common inherited condition that predisposes patients to colorectal cancer. The underlying cause of FAP is a germline mutation in the adenomatous polyposis coli (APC) gene on chromosome 5, which is involved in the adenoma carcinoma sequence [37,38,39]. This gene codes for the tumor suppressor protein APC, which regulates the growth and division of cells through regulation of the Wnt signaling pathway [39,40]. Mutations in this gene can result in impaired beta-catenin phosphorylation and hence decreased degradation of beta-catenin and result in abnormal cellular survival and proliferation [40]. Mode of inheritance for APC mutations is autosomal dominant; although de novo mutations are also common [31]. This is highlighted by the fact that about one third of FAP patients do not have an affected parent; but can pass the new mutations to their children [38]. Similar to Lynch Syndrome, FAP has extracolonic manifestations and involves the development of polyps in the stomach, fundus, and duodenum in early adolescence [41]. These polyps are at increased risk for malignancy. If patients with FAP are left untreated, polyps may develop into cancer [38]. 

##### Patient Specific Risk Factors

The risk for colorectal cancer carcinogenesis increases after the age of 40, and progressively increases from there, with a sharp rise after the age of 50. The incidence rate is 50 times higher in people aged 60–79 compared to those less than 40 years old [32]. Thus, screening is recommended in patients greater than 50 years old, unless other risk factors are present requiring earlier screening [42]. In the United States, 19% of people may develop adenomatous polyps, which are considered a precursor to colorectal cancer carcinogenesis. Thus, a personal history of adenomatous polyp development increases the risk of colorectal cancer carcinogenesis [32].

Inflammatory bowel disease (IBD) is considered as another risk factor for CRC. IBD consists of both ulcerative colitis and Crohn’s disease. 7-14% of people with IBD have been reported to develop CRC after having the disease for 25 years [30]. Individuals with IBD must undergo more colorectal cancers screenings to detect carcinogenesis early [32]. The increased risk of CRC in IBD could be attributed to the continued inflammation and increased permeability in the colon cell wall.

Diets that contain large amounts of animal fat also have a strong correlation with CRC development [32]. It is hypothesized that such diets promote bacterial flora that degrade bile salts into carcinogenic *N*-nitroso compounds [32,43,44]. Additionally, production of compounds with carcinogenic properties such as heterocyclic amines and polycyclic aromatic hydrocarbons, upon cooking meat with high temperatures, is also linked to CRC. Changes in diet can reduce up to 70% of an individual’s risk for developing colorectal cancer [32]. Other research suggests that differences in the amount of dietary fiber may account for differences in CRC incidences [32,45]

Colorectal cancers have been linked to the amount of physical activity and their overall body mass index (BMI) [32,46]. Frequency and intensity of physical exercise is inversely related to the incidence of colorectal cancer [32]. Smoking also causes harm to the colon and rectum, in addition to the lungs. Twelve percent of colorectal cancer deaths are linked to the carcinogenic effects of tobacco, which increase neoplasm growth in the colon and rectum. Long-term cigarette smoking causes more adenomatous polyp development and is associated with a younger average age of colorectal cancer carcinogenesis and larger polyps [32]. 

Excessive alcohol consumption is also associated with a younger age of onset of colorectal cancer carcinogenesis, because reactive metabolites of alcohol, like acetaldehyde, are carcinogenic [32]. Excess alcohol can increase tissue permeability and diffusion of carcinogenic molecules into the colonic and rectal tissues. Patients, who drink excess alcohol, are also at risk for malnutrition, thus affecting their diet. The effects of alcohol are also due to free radical oxygen species generation, lipid peroxidation, and prostaglandins production. This is theorized to be due to the induction of cytochrome P-450 2E1 by chronic alcohol use [47].

**Table 1:** Summary of bacterial associations with colorectal cancer (CRC) or promotion of CRC.

Bacterial Associations with CRC			
Strain	Model/Samples	Key Findings/Associations	Possible Mechanism(s)
*Bacteroides fragilis*	Patient samples: Tissue [9], Stool [10] Murine model of microbe-induced colon tumorigenesis [12]	Increased abundance of ETBF in early-stage lesions [9] and stool samples [10] *Bacteroides fragilis* toxin (BFT) mediated increase in IL-17 dependent NF-κB activation, chemokines production and myeloid cell accumulation [12]	BFT mediated tumorigenesis of colonic epithelial cells through mechanisms dependent on STAT3 activation, and IL-17 signaling mediated NF-κB activation, production of C-X-C chemokines, and recruitment of CXCR2-expressing myeloid cells [12]
*Streptococcus bovis/gallolyticus*	CRC tissues from patients with or without bacteremia [17] In vitro with human colonic epithelial cell line Caco-2; and rat model of azoxymethane-induced colon carcinogenesis [15]	Concomitant colorectal tumors present in about 25 to 80% of patients with *S. bovis/gallolyticus* bacteremia [14] *S. gallolyticus* isolated from 20.5% and 17.3% tumorous and non-tumorous tissues, respectively, from CRC patients with bacteremia compared to 12.8% and 11.5%, respectively, of CRC patients without bacteremia [17] Increased expression of IL-1, IL-8, and COX-2 in tissues from *S. gallolyticus*- positive CRC patients compared to the bacteria negative and control samples [17] *S. bovis* wall extracted antigens (WEA) increased release of CXC chemokines and PGE_2_ and increased aberrant crypt formation in vivo. In vitro, WEA increased IL-8 and PGE_2_ release as well as increased COX-2 expression and MAPK activation in Caco-2 cells [15]	Increased MAPK activation, bacterial dysbiosis, and overall increased inflammatory responses [14] *S. bovis* WEA promoted formation of pre-neoplastic lesions through mechanisms dependent on increased release of IL-8 and PGE_2_, increased expression of COX-2, and increased activation of MAPK signals [15]
*Fusobacterium nucleatum*	Stool samples from patients and healthy controls [16] In vitro cell line (HCT116), xenograft mouse model, and human colon specimens [18] Human NK cells, tumor-infiltrating lymphocytes (TILs), various human cancer cell lines, and colon carcinoma tissues [48]	Higher levels of *F. nucleatum* detected in patients with adenoma and CRC compared to healthy controls [16] Increased FadA (virulence and attachment factor of *F. nucleatum*) gene expression detected in colon specimens of patients with precancerous adenomas or CRC. FadA increased CRC cell proliferation. [18] Decreased NK cell killing of tumor cells and decreased activity of TILs upon Fap2 (virulence factor of *F. nucleatum*) binding to TIGIT [48]	FadA promoted the E-cadherin/beta-catenin-mediated proliferation of CRC cells in vitro and E-cadherin-mediated growth of CRC and expression of pro-inflammatory cytokines in vivo [18] Immune evasion mediated by inhibition of NK and T-cell anti-tumor activity upon Fap2 binding to the inhibitory receptor TIGIT [48]
*Enterococcus faecalis*	Retroscopic study of patients with *Enterococcus faecalis* infective endocarditis [49]	50.8% of patients with unknown source of E faecalis infective endocarditis were diagnosed with colorectal neoplasia upon colonoscopy [49]	
*Escherichia coli*	In vitro infection of murine enterocytes and colon carcinoma cell lines with *E. coli* [50] Human intestinal organoids exposed to polyketide-peptide genotoxin (Colibactin) expressing *E. coli* over a period of 5 months [51] Human monocytic THP-1 cell line differentiated into macrophages and infected with colon cancer-associated *E. coli* strain [52]	Infection of cells with polyketide-peptide genotoxin (Colibactin) expressing *E. coli* led to a significant increase in frequency of gene mutation and anchorage-independent colony formation [50] Exposure of intestinal organoids to colibactin-producing E. coli led to mutational signature which is similar to mutational structure found in two independent CRC cohorts [51] Survival of cancer-associated E. coli intracellularly in macrophages led to persistent increase in COX-2 expression [52]	Polyketide-peptide genotoxin-induced DNA double stranded breaks, incomplete DNA repair, and induced aneuploidy and tetraploidy [50] Colibactin dependent mutations likely through alkylation of DNA on adenine residues and subsequent double stranded DNA breaks [51] Infection by cancer-associated E coli. Increased COX-2 expression by macrophages in a p38 MAPK dependent manner [52]

MAPK: Mitogen-activated protein kinase, COX-2: Cyclooxygenase-2.

**Table 2:** Summary of bacterial associations with protection from CRC.

Bacterial Associations with Protection from CRC			
Strain	Model/Samples	Key Findings/Associations	Possible Mechanism(s)
*Bifidobacterium longum*	Feces from healthy persons taking or not taking *B. longum* and fructo-oligosaccharides (FOS); Human colon cancer cell lines [22] *B. longum* administration in colitis-induced murine model of CRC [23] Rat model of 2-Amino-3-methylimidazo[4,5-f]quinolone (IQ) induced colon cancer [24]	Increased amounts of short-chain fatty acids (SCFAs) and decreased *Bacteroides fragilis* enterotoxin in feces of individuals taking *B. longum* and FOS. In vitro, SCFAs, such as butyric acid, isobutyric acid, and acetic acid, had growth inhibitory activity against colon cancer cell lines [22] *B. longum* administration increased expression of tumor suppressor micro-RNAs (miRs) miR-145 and miR-155, decreased expression of miR-146a (regulator of interleukins 1β and 6), decreased nuclear factor kappa-light-chain-enhancer of activated B cell (NF-κB) activation and resulted in decreased aberrant crypt foci numbers [23] Dietary supplementation with *B. longum* led to 100% inhibition of IQ-induced incidence of CRC [24]	Ingesting *B. longum* with FOS leads to decreased *B. fragilis* enterotoxin, increased production of SCFAs, and subsequent inhibition of colorectal carcinogenesis and cancer cell growth [22] Decreased expression of oncogenic miRNAs and increased expression of tumor suppressor miRNAs [23]
*Lactobacillus*	Rat model of 1,2-dimethylhydrazine (DMH)-induced precancerous growths in colon [53] Murine model of azoxymethane (AOM)-induced colon cancer [54] Rat model of 1, 2-dimethylhydrazine (DMH)-induced CRC [55] Rat model of 1,2-dimethyl hydrazine (DMH)-induced CRC [56]	*Lactobacillus acidophilus* administration decreased aberrant crypts formation in colon [53] *Lactobacillus acidophilus* decreased incidence of colonic lesions by about 57% (compared to 27% by *Bifidobacterium bifidum*) [54] *Lactobacillus salivarius* Ren treatment led to 40% decrease in aberrant crypt foci formation [55] *Lactobacillus salivarius* Ren treatment led to significant decrease in cancer incidence compared to controls (from 87.5% to 25%). Administration of *Lactobacillus salivarius* Ren reduced *Ruminococcus sp*, *Clostridiales*, and *Bacteroides dorei*, and increased *Prevotella* [56]	*Lactobacillus acidophilus* administration decreased number of *E. coli* in feces, decreased activities of DMH metabolizing enzymes β-glucosidase and β-glucuronidase, and decreased plasma triglyceride concentration [53] *Lactobacillus acidophilus* administration significantly increased number of CD4^+^ and CD8^+^ T-cells [54] *Lactobacillus salivarius* Ren treatment increased SCFA levels and decreased azoreductase activity [55]
*Bacteroides fragilis*	Murine model of azoxymethane (AOM)/dextran sulfate sodium (DSS)-induced colitis-associated CRC [13] Human CRC cell lines in vitro [57]	B. fragilis colonization decreased DSS-induced inflammation and colitis, and decreased size and numbers of AOM/DSS-induced colitis-associated CRC tumors [13] *B. fragilis* Polysaccharide A (PSA), in TLR2 dependent manner, inhibited proliferation of CRC cells by suppressing cell cycle progression (downregulation of *CCND1* and *CDK2*, upregulation of *CDKN1B*). PSA suppressed EMT and decreased migration and invasion of CRC cells in vitro [57]	Protection against CRC was dependent on *B. fragilis* polysaccharide A production and toll-like receptor 2 (TLR2) signaling and associated with inhibition of C-C motif chemokine receptor5 (CCR5) in colon [13] Inhibition of cell cycle progression and inhibition of EMT and cancer cell migration and invasion in TLR2 dependent manner [57]
*Clostridium*	Murine model of 1,2-dimethylhydrazine dihydrochloride (DMH)-induced CRC and human colon cancer cell lines [58] Murine model of high-fat diet (HFD)-induced intestinal tumor [59]	*Clostridium butyricum* decreased DMH-induced colon cancer incidence from 90% to 30% [58] Oral administration of *Clostridium butyricum* led to significant decrease in numbers of HFD-induced intestinal tumors [59]	*Clostridium butyricum* inhibited proliferation of colorectal cancer cells, increased cell-cycle arrest and apoptosis of colon cancer cells, and modulated T-cells [58] *Clostridium butyricum* increased SCFAs and G-protein coupled receptor GPR43, suppressed tumor cell proliferation, increased tumor-cell apoptosis, and suppressed the Wnt/β-catenin signaling pathway [59]

#### 1.2.2. Therapeutic Targets in Colon Cancer

Colon cancer therapy is a rapidly evolving field. Many therapeutic targets have been established while others are still under investigation. Below, we briefly discuss possible targets that might be associated with or affected by probiotics and bacteria. 

##### Cytokines

Inflammatory cytokines in colon cells such as interleukins 6 and 17 (IL-6, IL-17) are involved in human colonic carcinogenesis. They have been shown to be increased in patients with CRC [60,61]. They induce the oncogenic STAT3 pathway and activate proliferative, anti-apoptotic, and pro-carcinogenic genes involved in cancer growth [12,61,62]. The essence of the inflammatory process related to cytokines is based on human colonic bacteria. For example, enterotoxigenic *Bacteroides fragilis* (ETBF), which secretes the *B. fragilis* toxin, triggers colitis and induces proinflammatory cytokines such as IL-17 to activate colonic neoplasia [61]. The use of probiotics can provide a stable equilibrium of gut flora by diversifying the microbiota population, which will in turn reduce toxins secreted by overpopulated *B. fragilis* [60,61].

Interleukin-23 (IL-23) may be involved in autoimmune inflammatory diseases (i.e., colitis, IBD) and is crucial for carcinogenesis. Colonization with *Escherichia coli* can increase IL-23 release by immune cells and may lead to cancer cell proliferation [63,64]. IL-23 has been shown to promote tumor growth, increase angiogenesis, increase matrix metalloproteinase (MMP9; an enzyme for extracellular matrix degradation) production, and curb cytotoxic T-cell recruitments to the tumors [65]. Altered microbial composition can be reversed by using probiotics to reduce genotoxicity and bring balance to gut microbiota, which may lead to reduced risk of CRC development by reducing the production of cytokines that induce tumor-promoting inflammation [66].

Transforming growth factor beta (TGF-β) has an essential role in inhibiting cell proliferation along with controlling immune regulation and microenvironment. It aids in regulating cell growth, death, and motility. Interestingly, both loss of TGF-β signaling and overexpression of TGF-β have been associated with CRC development and metastasis. Studies suggest that while TGF-β may have a tumor suppressor role in early stages of neoplasia development, it tends to promote growth and metastases in the later stages [67,68,69,70,71,72]. Its role in cancer allows the abnormal cells to continue to prevail and grow. Growing evidence suggests that TGF-β is also regulated by the microbiota. *Clostridium* bacteria has been shown to produce short chain fatty acids (SCFAs) such as acetate, propionate, and butyrate can increase the expression of TGF-β by epithelial cells in the colon; however, the mechanism of this relationship is not well studied [71]. 

##### Prostaglandin G/H Synthase

Prostaglandin-endoperoxide synthase (PTGS) promotes cell proliferation and has been associated with CRC incidence [73,74]. It also plays a central role in pro-inflammatory responses. Chronic inflammatory reaction in colorectal cancer is due to overexpression of PTGS2 by *Streptococcus gallolyticus* member bacteria (SGMB), which is believed to disrupt normal gut microbiota [48]. Probiotics may help replenish and introduce a variety of microorganisms to the intestinal flora. 

##### Proto-Oncogene RAF

RAF genes are essential in the RAS–RAF–MAPK signaling pathway. Overactivation of this signaling network results in cell proliferation, differentiation, and survival [75]. Activating mutations in RAF have been linked to approximately 8–12% of metastatic CRC cases [75]. Microbial imbalance exhibits loss of commensalism and diversity. This disproportion induces inflammation and promotes overexpression of inflammatory cytokines, which in turn lead to DNA damage [8]. The process of cancer development passes through a cascade of events that involves activation of proto-oncogenes such as c-RAF [8,75,76]. Restoring symbiotic gut microbiota by using probiotics may reduce chronic inflammation, overexpression of cytokines, and activation of proto-oncogenes by regulating the expression or mutation of the gene. The use of probiotics may restore microbial imbalance by restoring the health promoting bacterial strains. 

##### Vascular Endothelial Growth Factor (VEGF)

This growth factor (GF) regulates vascular permeability and development [77]. Bacteria have been shown to up-regulate intestinal VEGF expression [78]. VEGF signaling in colorectal cancer cells can promote colorectal cancer migration and invasion [79]. It has been shown that gut microorganisms can trigger mucosal endothelial and mesenchymal cells to promote TLR-dependent angiogenic responses involving VEGF [80]. Probiotic usage may offset changes in gut microbiota composition and decrease the promotion of angiogenic response and proinflammatory factors.

##### Fibronectin

High expression of fibronectin (a glycoprotein for cell adhesion) is associated with cellular proliferation and poor prognosis in CRC [81]. This glycoprotein serves as a cell-to-cell adhesion molecule. It promotes fibroblast migration, macrophage function, and binding of pathogens such as bacteria to mammalian cells. In colorectal cancer, fibronectin facilitates cellular proliferation, adhesion, tumor cell migration, epithelial to mesenchymal transition (EMT), metastasis, and induction of immunosuppression [81,82]. Colonization of *S. bovis/gallolyticus* in colorectal tissues through fibronectin adhesion and collagen-binding can cause serious inflammatory response [14,83]. Escalation of the inflammatory response induces proinflammatory and angiogenic cytokines, leading to the development of colorectal cancer. Imbalance of the gut microbiota is promoted by over colonization of a specific commensal bacteria, which may be corrected by supplementing probiotics to restore diversity and abundance of gut flora [14,82,83].

#### 1.2.3. Current Treatment and Its Limitations

Treatment options for colorectal cancer depend on staging and localizations. Staging is based on the TNM system: T is the size and extent of the primary tumor; N is the extent that the cancer has spread to the lymph nodes; and M is whether the primary tumor has metastasized [84,85]. The initial treatment of colorectal cancer usually begins with surgical resection of the primary tumor(s) and associated lymph nodes (colectomy and/or lymphadenectomy) and an application of chemotherapy for stages III and IV of the disease [85]. 

Fluoropyrimidines (thymidylate synthase inhibitors) such as 5-Fluorouracil (5-FU), capecitabine, and floxuridine are used as first line monotherapy. They inhibit thymidylate synthase, resulting in decreased DNA replication and cell growth, and are generally used to treat a variety of cancers [86]. These must be administered with leucovorin to enhance fluorouracil efficacy by increasing its binding to the enzyme and are sometimes given orally with cytotoxic agents (oxaliplatin, a platinum agent or irinotecan, a topoisomerase I inhibitor) to treat colorectal cancer [87]. Primary chemotherapeutic regimens FOLFOXIRI (fluorouracil, leucovorin, oxaliplatin, and irinotecan) and FOLFIRI (infusional fluorouracil, leucovorin, and irinotecan) have significantly improved colorectal cancer treatment. The new chemotherapeutic regimens FOLFOXIRI and FOLFIRI, combined with new drugs such as cetuximab (epidermal growth factor receptor inhibitor), have improved the prognosis of recurrent or metastatic colorectal cancer patients by boosting response rates in clinical trials [85].

Some new drugs have recently been approved for the treatment of colorectal cancer and include epidermal growth factor receptor (EGFR) inhibitors [88]. Recently, aflibercept (a vascular endothelial growth factor B (VEGFB) inhibitor) was also approved for the adjuvant treatment of metastatic colorectal cancer [88,89]. 

Inhibitor of apoptosis protein (IAP) family is considered a candidate for a new therapeutic target for colorectal cancer. This family of proteins play a crucial role in the regulation of apoptosis [90]. Survivin, a member of IAP family of proteins, is an inhibitor of apoptosis; and it has been shown to be elevated in many cancer types including colon [90]. In a study looking at the gene expression of survivin in HT-29 colon cancer cells, it was found that those treated with exopolysaccharides (EPS) from *Lactobacillus* bacterium had decreased gene expression of survivin, which resulted in increased apoptosis [91]. MicroRNA (miRNA) is also being examined as a potential therapeutic target for the treatment of colorectal cancer. miRNAs are involved in tumor progression, growth, and metastasis [92]. Currently, different miRNAs are being used as biomarkers and for detection and prognosis. Whether this target has therapeutic potential is still being investigated [92,93]. 

Probiotics have been investigated as a novel therapy for the prevention of colorectal cancer. It has been suggested that probiotic metabolites and their molecular signaling cascades might be utilized as epigenetic therapeutic targets. Further initial evidence has suggested that probiotics might also be used to actively treat colon cancer due to the involvement of leptin and environmental factors affecting colon cancer carcinogenesis. Leptin is a hormone, derived from adipose tissue, that helps to modulate different physiological processes (metabolic rate, reproduction, and immune response). Serum levels of leptin and expression of its receptor (LPR) are often altered in human colon tumors and leptin has been suggested as a risk factor for colon cancer [94,95]. Probiotic non-pathogenic bacteria consumption can decrease the expression of LPR, suggesting that probiotics may be the source of a novel therapy for colon cancer to improve survival rates [95]. 

Microsatellite instability (MSI), which is due to DNA mismatch repair (MMR), is usually not inherited. This occurs in around 15% of patients that get sporadic CRC [96]. It is also common, up to 70%, in the patients with loss of expression of MLH1 and PMS2 or with MLH1 methylation to have a BRAF V600E (a gene that increases cell growth) mutation [96]. Thus, germline testing is recommended if there is a strong family history of MSI. The MMR enzymes fix the errors that happen during DNA replication. If an individual has MMR deficiency (dMMR), the build-up of the errors during replication causes DNA to become unstable, resulting in MSI (microsatellite instability). MSI screening can identify tissues with a high amount of instability (MSI-H). The latest guidelines also include additional explanation of MMR immunohistochemistry testing for the four genes known to be mutated in Lynch Syndrome (*MLH1, MSH2,* MSH6, and *PMS2*) [96]. With regard to the treatments based on dMMR or MSI, different immunotherapy treatment options listed in the National Comprehensive Cancer Network (NCCN) guidelines for advanced or metastatic CRC are nivolumab (Opdivo), pembrolizumab (Keytruda), or a combination of nivolumab and ipilimumab (Yervoy), both in dMMR and MSI-H only. These recommendations are category 2B and are intended for patients who are not appropriate candidates for cytotoxic combination regimens. These same immunotherapy options are also listed in the guidelines as second- and third-line recommendations for dMMR/MSI-H patients [85]. MSI-tumors are more common in right sided CRC for an unknown reason. These tumors commonly use checkpoint regulators to remain alive and avoid the immune system; thus, checkpoint inhibitor immunotherapies such as pembrolizumab (a programmed death 1 inhibitor) have been shown to be efficacious [7]. 

Larotrectinib (Vitrakvi; tropomyosin kinase receptor inhibitor) is now a second-line treatment option for patients with metastatic CRC that is neurotrophic receptor tyrosine kinase (NTRK) gene fusion positive [97,98,99]. The gene fusion occurs when the NTRK 1/2/3 genes fuse with other genes, resulting in altered TRK protein (TRKA, TRKB, and TRKC) that activate signaling pathways for the proliferation of certain types of cancer. In the phase I trial, patients were given 50 mg of larotrectinib orally and were monitored for the primary endpoint of toxicities and safety. As a result, it was found that the drug was effective (in antitumor activity) and safe (minimal toxicities) [97]. The most common moderate toxicity that occurred was anemia, occurring in 6% of the 70 patients in the trial [97]. In further trials, the drug showed an overall response rate of 75% (complete response in 22% and partial response in 53%) [98]. In November 2018, the Federal Drug Administration (FDA) accelerated the approval of larotrectinib for the treatment of adult and pediatric patients with solid tumors, positive for a *NTRK* gene fusion without a resistance mutation [99]. The resistance mutation is found in the TRKA kinase domain; resistances are obtained by point mutations and include G623R, G696A, and F617L [97,98]. It is recommended in higher stages: tumors that are metastatic or where surgical resection is likely to result in severe morbidity, and when no satisfactory alternative treatments are available or that have progressed following treatment [99]. 

There are a variety of combination therapies that have been added to the guidelines as second line options. Some include dabrafenib (Tafinlar, BRAF inhibitor) plus trametinib (Mekinist, MEK inhibitor) plus cetuximab or panitumumab (mAB inhibitors of EGFR), another option is encorafenib (Braftovi; BRAF inhibitor) plus binimetinib (Mektovi; MEK inhibitor) plus cetuximab or panitumumab (EGFR inhibitor monoclonal antibody) [100,101,102]. These studies show that new therapies are arising and that patient factors (genetics, mutations, and resistance mechanisms) should be considered for determination of optimal treatment options. 

### 1.3. Probiotics

According to the Food and Agriculture Organization of the United Nations (FAO) and the World Health Organization (WHO), probiotics are “live microorganisms, conferring health benefits on the host when administered in adequate amounts” [103]. So, probiotics are live bacteria that can be added to any human diet as supplements that can potentially confer health benefits. Higher order vertebrates have been in a symbiotic relationship with microorganisms for millions of years on Earth, effectively co-evolving together. 

Humans are no exception to this relationship as it is estimated that 100 trillion different bacteria divided into 1000 different species inhabit the human gastrointestinal tract. The human gut microbiome is believed to have around three million functional genes compared to 23,000 genes in human beings; this far larger genome of the microbiome has correspondingly greater functional capabilities in modulating human physiology [104]. The human microbiome is now considered a fully functional additional organ that is highly adaptable, flexible, and organized with key functions for human health [103,104].

Humans acquire unique microbiota concentrations during natural processes at the inception of life, beginning with the passage through the birth canal and continuing via breastfeeding [105]. Different modes of child delivery have been shown to lead to different compositions of human microbiota: vaginally delivered babies have higher concentrations of *Bifidobacterium* than babies delivered via C-section [105] Infants who are breastfed also have higher concentrations of *Bifidobacterium* compared to those who are formula-fed; formula-fed babies have a higher concentration of *Enterococci*. There have been some associations with different disease susceptibility based on this early difference in microbial colonization. Beyond the effects of birthing method(s) and childhood diet, research has shown different microbiota arise from different kinds of diets in more mature humans. For example, rural African children have higher *Bacteroides*, lower *Firmicutes*, and display lower incidence of irritable bowel disease (IBD) when compared to European children with typical Western diets [106]. 

#### 1.3.1. History and Rationale behind the Use of Probiotics in Cancer

Genetics and environmental factors are the two main contributing factors to CRC. Other risks include IBD and the microbial composition of the intestines. Microbiota compositions have been shown to affect the following processes: epithelial cell proliferation and differentiation, production of bioactive food products and nutrients, prevention of overgrowth of pathogenic organisms, and stimulation of immunity (refer to Table 3 for a summary of the roles played by healthy human microbiota). To a large extent, the exact mechanisms by which the composition of microbiota are linked to CRC are still unknown. At present, studies have discovered evidence that normal microbiota are composed of both beneficial and pathogenic bacteria. If the pathogenic bacteria grow too rapidly, an inflammatory process can be triggered, resulting in the production of carcinogenic compounds. It is vital to recognize the role that healthy flora plays in protecting us against detrimental health conditions. Bacteria in our gut compete with potential invaders for space and nutrients as well as produce bacteriocins, which act as antibacterials to eliminate harmful bacteria from our intestines [102,104,105,107,108,109,110]. Thus, it is important that the balance of the normal gut floral remains at homeostasis.

Dysbiosis, microbial imbalance in gut or malabsorption in our body, can be caused by environmental factors (such as diet, infection, and antibiotics). Tackling dysbiosis and the effects of harmful bacteria with replacement through the use of probiotics results in protection against CRC or therapeutic response to different drugs in CRC. According to a murine study on the influence of gut microbiota dysbiosis to the efficacy of 5- Fluorouracil (5-FU), initial gut microbiota community composition is the key factor driving host response to the antitumor drug of 5-FU, which helped in exploring the potential probiotics may possess to enhance the efficacy of antitumor drugs [8,111]. Probiotics is a term used to describe healthy bacteria that have a beneficial effect on the body; lactic acid producing bacteria (LABs) such as *Lactobacillus* and *Bifidobacterium* species are the most common types that are found in the gut [109]. In the study by Yuan et al., probiotics with these species were used to show the results in increased response to the drug [111]. Probiotics maintain gut integrity, regulate bowel movement, improve lactose intolerance, thereby improving immunity and helping prevent harmful bacterial overgrowth and yeast infection. Probiotics are different from prebiotics. Prebiotics are carbohydrates that act as fuel for probiotics and are non-digestible in the human body. They support the growth or activity of the probiotics [112,113]. Both probiotics and prebiotics (used together in synbiotics) can aid in the prevention of dysbiosis. 

It is important to understand the key players involved in maintaining the gut microbiota balance because it can potentially be prophylactic against CRC. Several studies have documented differences in microbiota composition between healthy individuals and patients with CRC [108,112]. One study found that the stool of CRC patients contained more *Bacteroidetes/Prevotella* when compared to healthy patients [114]. The core human colonic commensal microflora is composed primarily of *Firmicutes*, *Bacteroidetes*, *Proteobacteria*, and *Actinobacteria* with concentrations a million-fold higher than in the small intestine. Even though there are broad similarities in the proportion of general categories of commensal microbes, there are significant differences between apparently healthy individuals at lower taxonomic levels. Abnormalities in the host microbiome composition and/or function are associated with the etiology of cancer pathogenesis [108,114].

Another study was conducted to find out the differences between the normal and colorectal cancer mucosa bacterial composition. This study by Gao et al. aimed to find out how probiotics affect the microbiota in colorectal cancer patients. The results of the study showed that patients with colorectal cancer have different bacterial composition compared to mucosa of the normal healthy control group [115]. The study also found that colorectal cancer mucosa showed significantly increased concentrations of *Fusobacterium*, *Selenomonas*, and *Peptostreptococcus* after probiotic use. Aside from this finding, a group of patients in the study who had colorectal cancer were given probiotics for almost a month and biopsies were collected just before they underwent surgery. The probiotics included live *Bifidobacterium longum, Lactobacillus acidophilus,* and *Enterococcus faecalis*. The biopsies all contained increased concentrations of butyrate-producing bacteria such as *Faecalibacterium* and *Clostridiales*, and decreased concentrations of *Fusobacterium* and *Peptostreptococcus*. *Peptostreptococcus* and *Fusobacterium* play a role in colorectal cancer pathogenesis and can therefore be used as biomarkers to detect the disease in early stages. Additionally, this study, as well as other literature, highlight how there is a difference in bacterial composition between healthy and CRC patients, and how supplementing the diet of CRC patients with probiotics can change their microbiota for clinical benefits [115,116]. 

**Figure 1:**
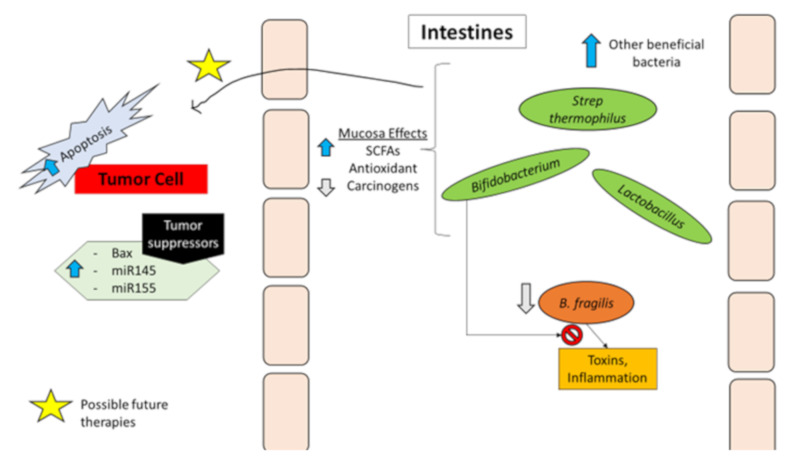
Mechanisms of probiotics. Probiotics increase other beneficial bacteria, while decreasing pathogenic bacteria and their harmful mechanisms. They have also been shown to increase short chain fatty acids (SCFAs) and antioxidants, while also decreasing carcinogens in the intestines. Possible actions that probiotics have are to increase tumor cell apoptosis and increase in tumor suppressor genes; these may be possible areas to research further into for colorectal cancer (CRC) prevention and adjuvant therapies.

Similarly, another paper highlighted the fact that CRC patients have an abundance of *Fusobacterium* [117]. This can be exploited to detect CRC polyps earlier than current diagnostic methods. With regard to the mechanisms through which *Fusobacterium* promotes carcinogenesis, it has been suggested that, due to its interaction with E-cadherin, it enhances the malignant potential of CRC by increasing inflammation and antagonizing the immune function of T cells. Another proposed mechanism is that *Fusobacteria* may promote colorectal cancer by activating Wnt/β-catenin signaling and damaging DNA ROS production and activation of oncogenes [117]. Others have reported enrichment of enterotoxigenic *Bacteroides fragilis* and *Enterococcus faecalis* in feces of patients with colorectal cancer compared to healthy controls [83,118]. *E. coli* has also been shown to promote the onset of CRC by expressing a polyketide synthase gene that is involved in inflammation, cell proliferation, and epithelial cell injury via a direct invasion of the epithelial cell layer [119]. The polyketide synthase gene also enhances the activity of cyclooxygenase 2 (COX-2), which is linked to CRC by several additional studies [116,119]. There are many toxins produced as a result of microbiota dysbiosis. One of those toxins is the *B. fragilis* toxin (BFT). BFT interacts with Wnt/B-catenin and nuclear factor kappa B at the molecular level; all three mediate inflammation and cell proliferation, thus contributing to carcinogenesis [8]. Toxins can also have directly damaging effects on DNA [8]. Another example is polyketide peptide toxin, which creates gene instability [50]. Additional mechanism involved in carcinogenesis when dysbiosis occurs is the fact that pathogenic bacteria contain enzymes such as beta-glucuronidase and azoreductase. These enzymes are capable of converting byproduct molecules from a person’s diet into carcinogenic substances [55]. Probiotics have been shown to decrease the secretion of these enzymes and limit this conversion of byproduct molecules into carcinogenic substances, thus potentially reducing rates of CRC occurrence [8,55,115,119]. Figure 1 contains a summary of the actions that probiotics have, along with possible therapeutic targets. 

#### 1.3.2. Types or Strains Used

Studies have revealed that the colorectal cancer microbiome differs from that of nearby normal tissue with diminished diversity and distorted community structure, lower relative abundance of potentially protective short chain fatty acid-producing bacteria, and increased abundance of taxa with potentially carcinogenic inducing phenotypes [114]. However, it is currently unclear whether this change in microbiome is a risk factor for colorectal cancer development or if it contributes to downstream pathogenesis signaling processes.

Lactic acid bacteria (LAB) are a highly common type of probiotic inhabiting human intestines, which have many useful functions. Amongst those functions is the antitumor property possessed by the exopolysaccharides (EPS) that are produced by lactic acid bacteria. EPS are polysaccharides that are located outside of the cell wall. A recent experiment examining the antitumor property of EPS involved obtaining human colon cancer cells HT-29 and inoculating with EPS obtained from different strains of *Lactobacillus* [91]. The study found morphological changes in the chromatin of cells that were inoculated with EPS and an anti-proliferation effect on H-29 cells in vitro [91]. It was also found that the inhibition is dependent upon dose with an optimal concentration of 500 mcg/mL of EPS being essential to maximize the anti-proliferation effect. The effect of EPS on cell cycle phase distribution and apoptosis was also examined. A higher percentage of cells treated with EPS were found to be in G0/G1 compared to S/G2. In addition to its effect on cell cycle phase distribution, EPS inoculation induced apoptosis in H-29 cells [91]. 

*Lactobacillus* and *Bifidobacterium* are among the most commonly studied bacteria that have promising properties for use in the treatment of colon cancer. These properties include anti-apoptotic, anti-proliferative, and antioxidant effects on cancer cells. It has been shown that lactic acid bacteria enhance the action of pro-apoptotic proteins involved in the cell cycle such as Bax, and they downregulate anti-apoptotic proteins such as Bcl-2 [120]. It is not currently understood which component or product of bacteria exerts these pro-apoptotic effects on cancer cells, but it has been hypothesized that it is due to short chain fatty acids (SCFA) produced by the bacteria, particularly butyric acid. SCFA are the products of bacterial fermentation of non-digestible carbohydrates [27]. Normal cells typically utilize butyric acid as a carbon source for energy production. It has anti-apoptotic effects on noncancerous cells and has been shown to prevent the proliferation of cancerous cells [121,122]. At the molecular level, butyric acid was noted to act as a histone deacetylase inhibitor in cancer cells leading to the acetylation of the pro-apoptotic genes, hence increasing their expression [122,123]. Butyric acid also decreases the expression of the genes for cyclin D1 (a regulator of cell progression in G1 and S phase of the cell cycle), which have been hypothesized as playing an essential role in CRC pathogenesis [124,125]. Histone deacetylase inhibitors (HDAC inhibitors) cause DNA strain and possible breakage in both normal (non-cancerous cells) and cancerous cells. However, normal cells can recover from this destruction and will repair themselves while the cancerous cells cannot [126]. Gut microbiota can contribute to HDAC inhibition through production of butyrate, valeric and hexanoic acid, which are thought to contribute to the HDAC inhibitory effects [127]. In addition, *Lactobacillus acidophilus* has been found to increase the pro-apoptotic effects of the typical anticancer treatment, 5-Fluorouracil, and it increases the expression of caspase-3, resulting in cell death [107]. Probiotics also stimulate the production of mucins in the intestines, which acts as a protective barrier against pathogenic bacteria [107,128]. SCFAs are also a source of energy that aids in the proliferation of healthy cells [122]. Due to these qualities, the adequate production of SCFA is an important indicator of healthy microbiota. Appropriate diet consumption plays an important role in determining the presence of symbiotic microbiota that carry out many important functions including the fermentation of food products and the subsequent production of beneficial SCFAs [107,122].

Another study was conducted to investigate the effect of probiotics on the production of SCFA and the composition of the intestinal flora (such as putrefactive bacteria) and *Bacteroides fragilis* enterotoxin (ETBF) [24]. SCFAs such as butyric acid exerts growth inhibitory and pro-apoptotic effects on cancer cells [24,107,129]. In the study by Ohara et al., the researchers attempted to directly examine the role of probiotics and prebiotics in colon cancer prevention by directly measuring the amount of SFCA, putrefactive bacteria, and ETBF in human fecal samples [24]. After extraction of these samples, human colon cancer cell lines were subjected to those SCFAs to test for anti-proliferative properties. For human study, subjects were divided into two groups: (1) was given yogurt containing only *Bifidobacterium longum* (a probiotic), and (2) was given both Bifidobacterium longum as well as fructo-oligosaccharides (a prebiotic). The results of the study showed that ETBF and fecal putrefactive products were significantly reduced after five weeks of consuming probiotics. Additionally, SCFA production was increased (especially butyric and isobutyric acid) in both groups with the introduction of probiotics and prebiotics into the subject’s diets [24].

Another proposed mechanism is the prevention of conversion of procarcinogens to carcinogens by the enzymatic detoxification and biotransformation carried out by strains of *Lactobacillus* and *Bifidobacterium* [130]. For example, L. *rhamnosus* has been shown to bind to *N*-methyl-*N’*-nitro-*N*-nitrosoguanidine (a carcinogen and mutagen), leading to biotransformation and detoxification. In vivo in rats, oral administration of *L. rhamnosus* led to protection against *N*-methyl-*N’*-nitro-*N*-nitrosoguanidine induced damages [131]. In addition, certain strains like *L. acidophilus* have been shown to decrease the activity of the enzymes beta-glucuronidase, azoreductase, and nitroreductase [131]. These enzymes have been shown to be involved in tumor formation; they convert procarcinogens into carcinogens. For instance, they act on polycyclic aromatic hydrocarbons, heterocyclic aromatic amines, and primary bile acids to convert them into carcinogens [131]. 

Another proposed mechanism is immunomodulation through the production of anti-inflammatory cytokines [132,133]. Probiotics have been shown to increase the production of anti-inflammatory cytokines, increase phagocytosis by macrophages, and increase tumor cell apoptosis. Probiotics have been found to have these effects to some degree, resulting in lower tumor progression [132,133]. Probiotics are also involved in maintaining an adequate intestinal pH level. Elevated pH and decreased SCFAs have been found in the faeces of patients with CRC, suggesting an inverse relation between SCFAs and pH [134].

As above-mentioned, it is not only the probiotic itself that is useful, but the metabolites that they produce. Nisin is a bacteriocin produced by *Lactococcus lantis*, which has been shown to have anti-metastatic effects on cancer cells. A study was conducted to evaluate the effect of nisin on the expression of genes involved in metastasis such as CEA, CEAM6, and MMP2F [135]. These genes are linked to unregulated growth, immune evasion, metastasis, and resistances. This study revealed that nisin reduced the expression of all of these genes as well as decreased the protein expression of carcinoembryonic antigen (CEA) [135]. 

#### 1.3.3. Current Products on Market

Commercial probiotics use different strains of bacteria such as *Lactobacillus rhamnosus, Bifidobacterium longum,* and *Bifidobacterium lactis* [136]. Kefir, a fermented milk probiotic, can contain *Streptococcus*, *Lactococcus*, *Lactobacillus*, *Leuconostoc*, *Bifidobacterium bifidum*, and *Acetobacter* species and also some yeast species (Saccharomyces, Kluyveromyces, and Candida) [137]. It has been proven to have powerful anticancer properties. At the molecular level, Kefir contains bioactive peptides that are involved in the activation of macrophages and enhancing phagocytosis. Kefir also increases the production of anti-inflammatory cytokines such as IL-4 and IL-10, and also leads to an increased IgA secretion, improving mucosal immunity and decreasing inflammation [137,138]. In addition, it has been shown to decrease the expression of anti-apoptotic gene Bcl-2 while increasing the expression of the pro-apoptotic gene Bax in gastric cancer cells [137,138]. Kefir also reduces DNA damage through its anti-oxidative properties, which include increasing the level of glutathione peroxidase and decreasing the level of malondialdehyde [138]. A study has shown that the supernatant of kefir contains lactic and acetic acid with antioxidant properties reducing DNA damage and causing G1 cell cycle arrest in colorectal cancer cells [137,138].

VSL#3 (Value of Statistical Life) packets is another probiotic formulation. It contains between 112.5 and 900 billion live colony forming units (CFU); it utilizes a combination of diverse strains of bacteria such as *Streptococcus thermophilus*, different types of *Bifidobacterial* such as *longum*, *infantis*, and *breve* [139]. VSL#3 also includes different types of Lactobacillus *(acidophilus, plantarum, paracasei, delbrueckii*). In a preclinical study, it was shown that VSL#3 administration led to decreased inflammation and delayed onset of colonic dysplasia and cancer [140]. However, in a study of azoxymethane induced colitis-associated CRC in mice by Arthur et al., it was determined that giving VSL#3 did not protect against inflammation and CRC tumorigenesis [141]. 

Culturelle capsules contain *Lactobacillus rhamnosus* with 10 billion CFUs per capsule, but also contain 200 mg inulin (a prebiotic) [142]. This is an example of synbiotics (prebiotics and probiotics together). The microorganism in this product (*Lactobacillus rhamnosus*) has shown in one study to alter fecal microorganisms and prevent increase of IL-2 in patients with colorectal cancer [143]. The possible outcomes of the decrease in IL-2 were not noted in the article. However, low levels may not have a beneficial effect on patients with preexisting CRC, since IL-2 helps to activate the immune system [143,144]. The study was most likely looking at the prevention measures that the probiotic could result in. Culturelle is also one of the top over the counter probiotics on the market. 

Activia yogurt contains *Bifidobacterium animalis* subsp *lactis* DN-173 010; with 100 million CFUs per gram [136,142]. This product has been studied in patients with colorectal cancer. It was found to decrease inflammation by decreasing nitric oxide and cytokine production [143]. Thus, it may be beneficial in the prevention and/or treatment of CRC. 

DanActive Cultured Milk is a probiotic product that contains *S. thermophilus* and *L. bulgaricus* in addition to *L. casei* DN-114 001. Each serving contains one billion CFUs per 3.1-ounce bottle [142]. This has not specifically been studied in colorectal cancer, but in a study on breast cancer in mice, it showed potential for anticancer actions [143]. 

#### 1.3.4. Clinical Trials

Current clinical trials have researched the benefits of probiotics. Studies, which were obtained online using PubMed and ClinicalTrials.gov, were mainly conducted on adults (greater than 18 years old) who had CRC. Many trials have studied non-metastatic cancer. Several have also attempted to reduce any bias in the study by excluding patients taking antibiotics or other prebiotics/probiotics/synbiotics in a certain timeframe. This was done to limit the impact these items would have on the gut microbiome. Some of the trials examined probiotic use and its effect on inflammatory factors (TRG1-2, cytokines) and mucosal proliferation. Results show these may be better markers to demonstrate benefits in CRC patients. Even though several studies may indicate clinical significance in the use of probiotics in the supplementation to CEC treatment as well as preventative measures, most state that further studies with larger cohorts are necessary to fully prove this theory. Clinical trials examined for this article are shown in Table 4 and Table 5. These trials show that probiotics aid in normal gut flora health, and they have effects on inflammatory factors and a possible role in colorectal cancer treatment. Many of these studies have not published their results separately.

## 2. Conclusions

Colorectal cancer is the third most common cancer worldwide. Current treatment options of surgical resection followed by chemotherapy have yet to be perfected or replaced with more targeted therapies. Gaining some understanding on the molecular pathways involved in the initiation and progression of colorectal cancer has been of paramount importance in designing treatment strategies that target certain pathways. Microbial metabolites like short chain fatty acids (SCFAs) have been shown to play pro-apoptotic, anti-proliferation, and anti-cancer roles. Their influence on epigenetics in terms of acting as histone deacetylase inhibitors and the subsequent activation of pro-apoptotic genes can be exploited in such a way that it can be used as primary or adjuvant therapy to current therapies available. Aside from the molecular pathways involved, it is also important to understand the ideal composition of microbiota that would serve to protect against colon cancer. Certain bacteria, like *Fusobacterium* and *Peptostreptococcus*, have been shown to be linked to the pathogenesis of colon cancer whereas *Lactobacillus* and *Bifidobacterium* have been shown to be cancer protective. Understanding how lifestyle modifications can influence this composition early on and educating the public can be of paramount importance in cutting down the incidence of colorectal cancer. However, more research is needed to provide us with clear-cut evaluations of the anti-carcinogenic, anti-inflammatory, and immunomodulatory properties of probiotics and the exact composition of microbiota that would support a healthy mucosa and maintain immune status to protect against inflammatory processes.

## Figures and Tables

**Table 3:** Mechanisms the probiotics can have on the body.

Nonspecific Physiological Mechanisms	Immunological Mechanisms
Initiate antiproliferative and apoptotic signaling in colorectal cancer cells	Modulate immune functions in gut mucosa
Bolster the intestinal mucosal barrier function	Induce natural killer cells
Inhibition of the enzymatic activity of pathogenic bacteria	Helps in immune maturation and maintenance
Inhibition of carcinogenic agents	Diversified gut flora positively modulates T-regulatory cells against tumor cells

**Table 4:** Clinical trials on probiotics in cancer.

Study Title	NCT #	Status *	Intervention	Study	Inclusion/Exclusion Criteria	Outcomes	Results	Published *
An Evaluation of Probiotic in the Clinical Course of Patients with Colorectal Cancer [145]	03782428	C	Probiotics (*Lactobacillus, Bifidobacterium*). Placebo	52 patients Double blinded, randomized Duration: 6 months	>18 years old, non-pregnant/nursing, with CRC planned for resection. No antibiotic or pro/prebiotic use in past 2–4 weeks	Level of circulating inflammatory cytokines. Episodes of chemo-induced diarrhea.	Decrease in inflammatory cytokines (ILs) 4w after surgery. Modified micro-environment	Yes
Probiotics in Colorectal Cancer Patients [146]	00936572	C	Probiotics (La1, BB536). Placebo given twice a day. Given for 3 days preoperatively	31 patients Triple blinded, randomized Duration: 1 year	18–80 years old going for colorectal surgery, able to provide fecal sample after pre-operatively. No immunological disorders.	Microbiology of gut flora and gastrointestinal function	La1 effects intestinal microbiota & decreases pathogenic bacterial concentrations. Little effect from BB536.	Yes
Using Probiotics to Reactivate Tumor Suppressor Genes in Colon Cancer [147]	03072641	C	ProBion Clinica (*Bifidobacterium, Lactobacillus*, Inulin)	20 participants Randomized Duration: 6 years	1+ malignant tumor in colon. No recent antibiotics or probiotics	Microbiology of gut flora after surgery. Genetic expression after probiotics.	Unknown	No
Prevention of Irinotecan Induced Diarrhea by Probiotics [148]	01410955	C	Probiotics (Colon Dophilus™). Placebo	46 patients Quadruple blinded, randomized Duration: 1 year	Life expectancy >3 months with CRC (with irinotecan therapy). No history of ileostomy, no active infections, no antibiotics	Incidence of diarrhea	Decrease in severe diarrhea episodes and decrease of diarrhea episodes. No infections caused by probiotics observed.	Yes
The Effects of Metchnikoff Probiotics on Symptom and Surgical Outcome [149]	03531606	C	Metchnik-off (Probiotics). Placebo	68 patients Randomized Duration: 2 years	>20 years old with sigmoid CRC. No metastasis, no preoperative chemo/radiotherapy. No use of pre/probiotics in 7 days	Resection improvement (by questionnaire)	Unknown	No
Synbiotics and Gastrointestinal Function Related Quality of Life After Colectomy for Cancer [150]	01479907	C	Synbiotics. Placebo Given postoperative.	100 patients Quadruple blinded, Randomized	CRC non-hereditary, non-metastatic. No history of IBD. Not pregnant patients.	Quality of life related to GI function ‘	Better quality of life score over 3 months. Less episodes of diarrhea. Non-significant for constipation episodes.	Yes
Gut Mucosal Microbiota is Associated with Colorectal Cancer Relapse [151]	03385213	C	No intervention post CRC treatment, including surgery	200 patients Case Control, retrospective Completion: December 2022	18–75 years old, normal weight (BMI 18.5–23.9 kg/m^2^) with CRC. No renal/liver impairment. No antibiotics or probiotics within 3 months. No history of IBD or chronic diarrhea	Microbiology of gut. Genetic expression changes.	Unknown	No
Influence of Sundilac and Probiotics on the Development of Pouch Adenomas in Patients with Familial Adenomatous Polyposis [152]	00319007	U	Sundilac. Probiotics (VSL #3). Prebiotic (Inulin)	30 patients Randomized Duration: 9 months	Proven FAP, restorative proctocolectomy with ileal pouch anal anastomosis. No renal/liver impairment, no history of ulcers, no aspirin within 3 months. No probiotics	Mucosal proliferation	Non-significant decrease in cell proliferation in any groups.	Yes
The Effects of Probiotics on Bowel Function Restoration After Ileostomy Closure in Patients with Rectal Cancer [153]	02751736	U	Probiotics (CJLP 243). Placebo Given daily for 3 weeks before & after ileostomy surgery.	40 patients Quadruple blinded, randomized Duration: 1 year	20–75 years old with CRC lower anterior resection, non-metastatic. Not pregnant, no valvular heart disease	Bowel function. MSKCC & LARS questionnaire scores.	Non-significant effect on improving bowel function. Non-significant use of questionnaires	Yes
VSL #3 Versus Placebo in Increasing the Pathological Major Response Rate in Patients with Rectal Cancer	01579591	U	Probiotics (VSL#3). Placebo	160 patients Double blinded, randomized Duration: 1 year	>18 years old with CRC, expected to live >6 months. No antibiotics or probiotics in 1–2 weeks to registration	TRG1-2 rate. SCFA expression. Adverse effects. Immune system changes.	Unknown	U
Intestinal Microflora in Colorectal Cancer (CRC) After Chemotherapy	02169388	U	Probiotic (*Clostridium Butyricum*). Placebo. Given twice a day for 4 weeks.	30 patients Triple blinded, randomized Duration: 4 months	18–80 years old, non-pregnant/lactating scheduled for chemotherapy. No renal/liver impairment. No use of antibiotic or pre/probiotics for 1 month	Microbiology and SCFAs in feces, adverse reactions during chemotherapy	Unknown	U
Lactobacillus Rhamnous in Prevention of Chemotherapy-related Diarrhea [154]	00197873	U	Probiotic (*Lactobacillus Rhamnosus*). Placebo. During chemotherapy.	84 patients Quadruple blinded, Randomized, Cross over Duration: 12 years	>18 years old with CRC expected to live >3 months. No diarrhea, no treatment with bevacizumab (within 12 months)	Number of bowel movements. Chemotherapy tolerability.	Unknown	U

* Status: Unknown (U), Completed (C), Recruiting (R).

**Table 5:** Ongoing/recruiting clinical trials for probiotics.

Study Title	NCT #	Status *	Intervention	Study	Inclusion/Exclusion Criteria	Outcomes	Expected End Date
Chemotherapy w/wo WeiLeShu in Metastatic Colorectal Cancer [155]	04021589	R	Probiotics (WeiLeShu™ from Tongchuang Biotechnology) with Chemotherapy	50 patients Randomized, Open label. Phase II	>18 years old with CRC with good renal function (creatinine > 2.9 mg/dl)	Progression free survival	July 2022
Prebiotics and Probiotics During Definitive Treatment with Chemotherapy-radiotherapy SCC of the Anal Canal (BISQUIT) [156]	03870607	R	Synbiotics. Normal nutrition.	75 patients Double blinded, randomized	>18 years old with squamous CRC, non-metastatic. No infections requiring antibiotics	Response to chemotherapy	February 2024
Effect of Probiotics Supplementation on the Side Effects of Radiation Therapy Among Colorectal Cancer Patients [157]	03742596	R	Probiotics. Placebo	40 patients Quadruple blinded, randomized Phase II	35–65 years old with stage I–III CRC. No antibiotic or pre/probiotic use recently.	Level of immunoglobulins (A, F, M), interleukins (6, 1, 1), tumor necrosis factor, C-reactive protein	December 2022
Probiotics as Adjuvant Therapy in the Treatment of Metastatic Colorectal Cancer [158]	03705442	R	Omni-Biotic 10. Loperamide. Given twice a day for 84 days.	76 patients Assessor blinded, randomized. Phase II	> 18 years old with mitotic CRC (with FOLFIRI), not terminally ill (<6 months to live), not using probiotics	Incidence of diarrhea	February 2020

* Status: Unknown (U), Completed (C), Recruiting (R).
